# Decision-making in closure of oroantral communication and fistula

**DOI:** 10.1186/s40729-019-0165-7

**Published:** 2019-04-01

**Authors:** Puria Parvini, Karina Obreja, Amira Begic, Frank Schwarz, Jürgen Becker, Robert Sader, Loutfi Salti

**Affiliations:** 1 Department of Oral Surgery and Implantology, Carolinum, Goethe University, Frankfurt, Germany; 2 Department of Oral Surgery, Universitätsklinikum Düsseldorf, Düsseldorf, Germany; 3 Department for Oral, Cranio-Maxillofacial and Facial Plastic Surgery, Medical Center of the Goethe University Frankfurt, Frankfurt am Main, Germany

**Keywords:** Oroantral, Fistula, Flaps, Grafts, Maxillary sinus, Complication management, Oral surgery, Decision, Oroantral communication

## Abstract

After removal of a dental implant or extraction of a tooth in the upper jaw, the closure of an oroantral fistula (OAF) or oroantral communication (OAC) can be a difficult problem confronting the dentist and surgeon working in the oral and maxillofacial region. Oroantral communication (OAC) acts as a pathological pathway for bacteria and can cause infection of the antrum, which further obstructs the healing process as it is an unnatural communication between the oral cavity and the maxillary sinus. There are different ways to perform the surgical closure of the OAC. The decision-making in closure of oroantral communication and fistula is influenced by many factors. Consequently, it requires a combination of knowledge, experience, and information gathering. Previous narrative research has focused on assessments and comparisons of various surgical techniques for the closure of OAC/OAF. Thus, the decision-making process has not yet been described comprehensively.

The present study aims to illustrate all the factors that have to be considered in the management of OACs and OAFs that determine optimal treatment.

## Background

Oroantral communication (OAC) acts as a pathological pathway for bacteria and can cause infection of the antrum, which further obstructs the healing process as it is an unnatural communication between the oral cavity and the maxillary sinus. The oroantral fistula (OAF) develops if the OAC remains open and becomes epithelialized. The oroantral fistula has its origin either from iatrogenic complications or from dental infections, trauma, radiation therapy, or osteomyelitis [1].

Clinical decision-making determines the optimal strategy in a particular clinical situation. Consequently, it requires a combination of knowledge, experience, and information gathering. Previous narrative research has focused on assessments and comparisons of various surgical techniques for closure of OAC/OAF [2]. Thus, the decision-making process has not yet been described comprehensively.

Clinical decision-making in closure of an OAC/OAF depends on multiple factors that include the size of the communication, time of diagnosis, presence of infection, and clinician’s experience. Moreover, the selection of management strategy is influenced by the quantity and quality of tissue available for closure of OAF/OAC and the potential placement of dental implants in the future [3]. The method presented is decision tree design. This approach enables to recognize uncertainty in clinical diagnosis and therapeutic decisions and hence develop strategies to manage these uncertainties. The present study aims to illustrate all the factors that have to be considered in the management of OACs and OAFs that determine optimal treatment.

## Etiology

**Fig. 1:**
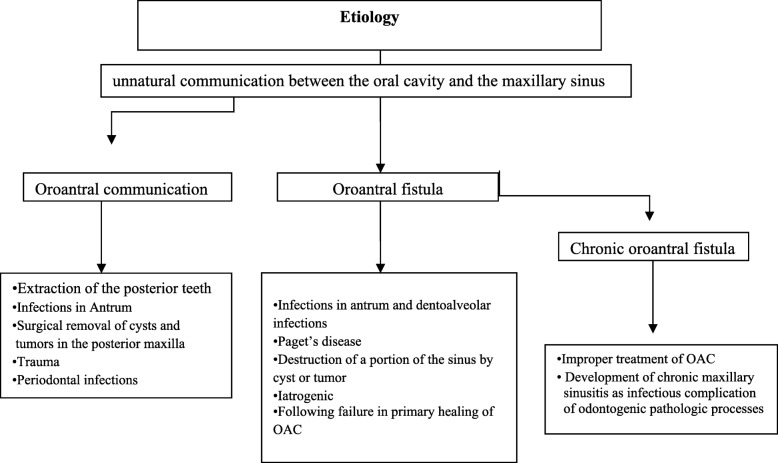
Represents etiology of OAC, OAF, and chronic OAF

Identifying the etiology of the OAC is essential to create an effective procedure. Harrison demonstrated that the bone lamella between the maxillary posterior teeth and the maxillary sinus is occasionally 0.5 mm [4]. Thus, the first premolars accounted for 5.3% of OACs, the second molars were the most frequently with an incidence of 45%, followed by the third molars 30% and the first molars 27.2%. It was reported that about 2.2% of the first molars apices perforated the maxillary sinus floor, followed by the second molars 2% of the described cases [4]. Due to the close relationship of the roots to the antrum and partially very thin maxillary sinus floor, the extraction of the upper molars and premolars, especially the extraction of the first molars, is considered the most common etiology of OAC [5–7]. Pathological lesions in the sinus, trauma, and failed external sinus floor elevation and augmentation can also lead to the formation of an OAC. Oroantral communication may be developed as a result of prevalence of the inflammatory odontogenic pathologic processes through the maxillary alveolar process to the Schneiderian sinus membrane. Periodontal infections and other factors are the least prevalent. Further complications of OAC may result from the removal of cysts or tumors, implant placement, maxillofacial surgery (Le Fort osteotomies), and pathological procedures like osteomyelitis. In addition to the size of the defect, possible maxillary sinusitis, odontogenic infections, cysts, tumors, foreign bodies in the maxillary sinus, and osteitis and osteomyelitis changes also likely play a crucial role in the formation of a chronic oroantral fistula. Furthermore, improper treatment of OAC can produce maxillary sinusitis and become chronic [8]. Figure 1 illustrates the etiologic factors of OAC/OAF/chronic OAF.

## Medical history

Medical history serves to identify patients who have a higher risk to develop complications during or after closure of OAC. Cardiovascular disease, diabetes, renal dysfunction, and hematological disorders may increase the risk of complications such as bleeding, infections, and delayed tissue healing [9].

## Signs and symptoms

The symptoms of an OAC can vary from purulent discharge through the fistula to the patient’s subjective feeling entry of oral liquids into the nostril on the same side of the maxillary [10]. The presence of one or more of the symptoms could be the indicator of an OAC or a fistula (acute, chronic). However, some patients may not present any of these findings if the perforation is too small or closed by a large polyp. Untreated defect can cause sinus contamination leading to infection, chronic sinusitis, and impeded healing [10]. A confirmatory and early diagnosis is therefore strongly recommended to permit successful closure.

**Fig. 2:**
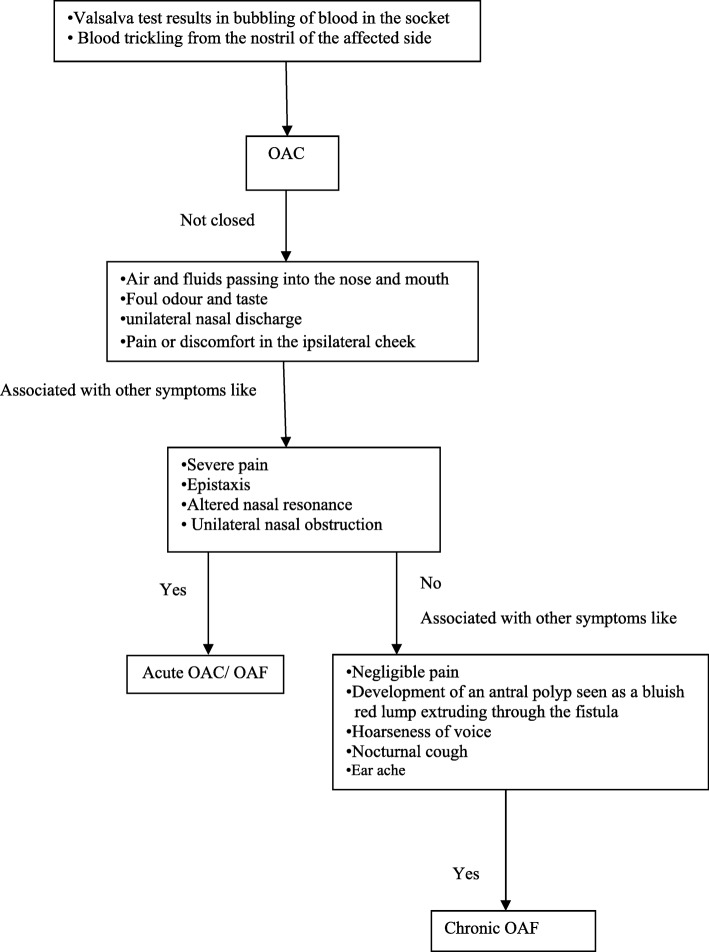
Illustrates steps of decision-making in symptoms of OAC, OAF, and chronic OAF

Figure 2 demonstrates symptoms based on whether the OAC is acute OAF or chronic OAF.

## Clinical examination and diagnosis

**Fig. 3:**
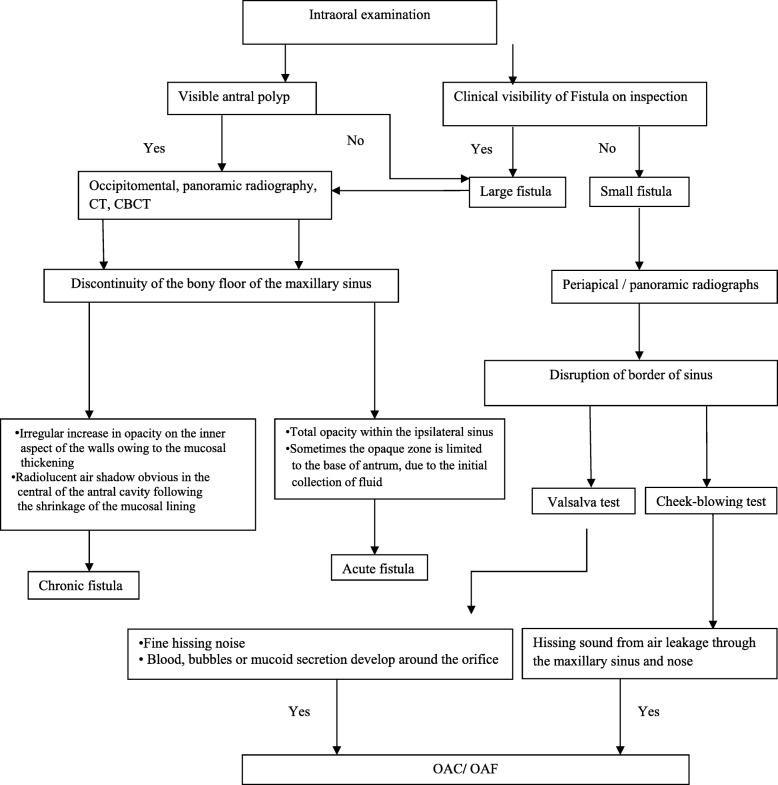
Illustrates steps of decision-making in diagnosis of antral perforation

Diagnosis represents the first decision-making about the patient. It determines all subsequent treatments and the course of each patient. It mainly based on a comprehensive evaluation of dental and medical examination and patient history, specifically looking for diagnostic criteria for maxillary sinusitis. Figure 3 illustrates the steps of decision-making in the diagnosis of antral perforation.

## Procedure

### Intraoral examination

**Fig. 4:**
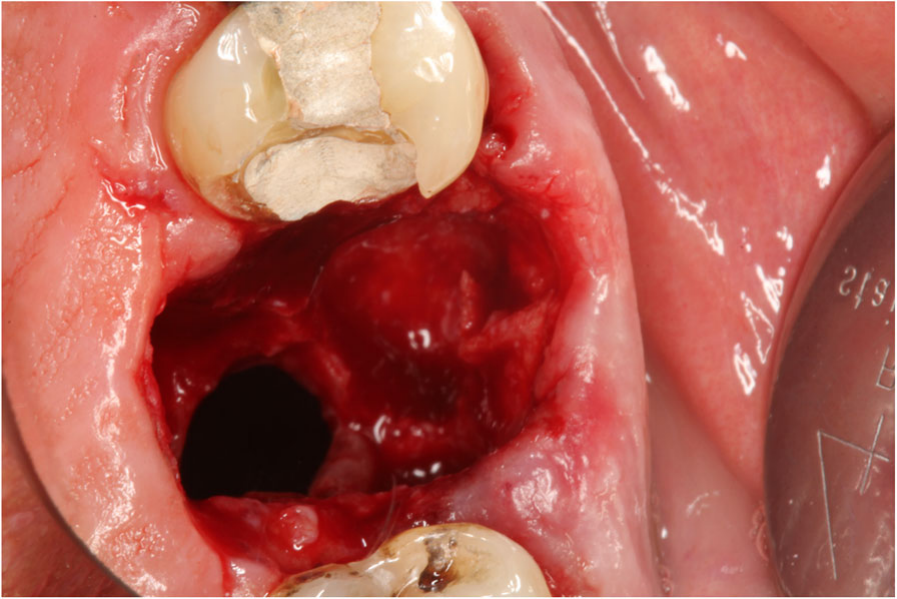
Clinical OAC after the extraction of a molar

The large OAC is easily seen on the investigation (Fig. 4). At a later stage, the antral polyp is seen through the defect.

### *Valsalva* test

The patient is instructed to try to exhale through a blocked nasal airway. However, a negative test does not exclude the possibility of antral perforation. It is worth noting that the detection of small perforations is not always possible [11].

### Cheek-blowing test

The patient is asked to blow air into the cheeks against a closed mouth. This test is considered a risk of antral complications due to the spread of microorganisms from the oral cavity into the maxillary sinus.

### Exploration of the perforation with probing

Attempt of probing the fistula is likely to result in sinusitis or widening of the fistula due to pushing of foreign bodies or bacteria into the maxillary sinus. [12]. Furthermore, probing may lead to laceration of the sinus membrane, which may sometimes be intact.

## Radiographic features of OAC and OAF

Radiological investigation of the site of OAC and OAF is required to validate the clinical findings and to investigate the presence of foreign body within the antrum. From an anatomical point of view, several different radiographic investigations are required to show all areas of the antral anatomy well because of the complexity of its anatomy [13].

Radiologically, bone discontinuity of the floor of the maxillary sinus is evident. Patients with OAF are most susceptible to sinus infections. Therefore, radiological investigation of the maxillary sinus is recommended. Periapical film or panoramic radiography can provide an idea about the bony defect size of the OAC and OAF. Radiologically, they reveal the disruption of the border of sinus. Periapical radiograph provides detailed information about the bony radiographic changes owing to its inherent technique quality. Moreover, it confirms the presence and location of the foreign body that may have been dislodged into the antrum [14, 15]. The maxillary sinus and the trajectory of the communication can be visualized by occipitomental and panoramic radiography. However, periapical film and panoramic radiography techniques give only a two-dimensional view of complicated three-dimensional (3D) structures. In addition, the structures are superimposed.

**Fig. 5:**
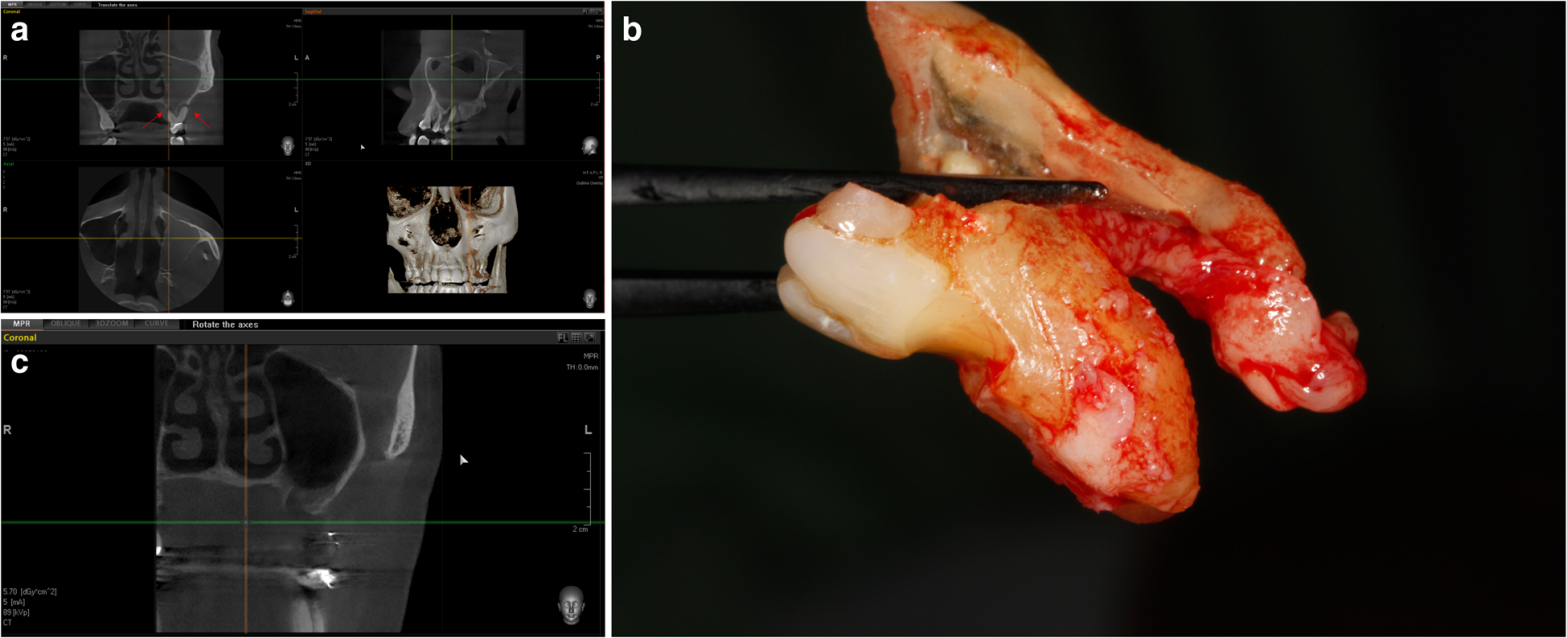
**a** CBCT of a molar with a periapical disease causing a maxillary sinusitis. **b** Extracted molar. **c** CBCT after a healing period of 3 months

Computed tomography (CT) and cone beam computed tomography (CBCT) scans are the gold standard modality of radiological assessment to rule out the presence of maxillary sinusitis [16]. Figure 5a shows a CBCT of a molar with a periapical disease causing maxillary sinusitis, Fig. 5b shows the extracted molar, and Fig. 5c shows a CBCT after a healing period of 3 months. Furthermore, both modalities can be used to assess the size of the fistula and to characterize the bone and mucosa surrounding the perforation and the nature of the sinus mucosal lesion [13, 16]. CT may reveal air-fluid interface, disruption of the floor of the antrum and foreign body. Figure 3 illustrates the steps of decision-making in radiographic diagnosis of antral perforation and the radiographic findings.

## Decision-making in treatment of OAC and OAF

The objective of the management of OAC/OAF is the closure of the defect and prevention of oral bacteria and food debris penetrating the sinus. Oroantral communication can cause sinus contamination leading to infection, impeded healing, and chronic sinusitis [10]. It is possible that a small OAC of less than 2 mm in diameter, without epithelialization and in the absence of sinus infection, can heal spontaneously after a blood clot is formed [17]. However, defects that are larger than 5 mm in diameter or those that present for more than 3 weeks rarely heal spontaneously and typically will require surgical intervention [18]. Technical choice of professionals for the closure of oroantral fistula can be influenced by the clinical aspects of each defect (location and size), further prosthetic treatment, and experience of surgeons [19]. Unilateral odontogenic sinus infection is treated and cured by drainage and removal of the odontogenic cause. Further factor is the outcome desired like the choice for bone or bone substitute grafting technique if the dental implant has to be placed in the near future. Moreover, in relation of OAC to adjacent teeth, the height of the alveolar ridge, duration of OAC, existence of inflamed sinus, and the general health of the patient should be taken into consideration [20]. The OAC must be closed within 24–48 h as its persistence increase the possibility of maxillary sinusitis [21].

## Preoperative procedures

**Fig. 6:**
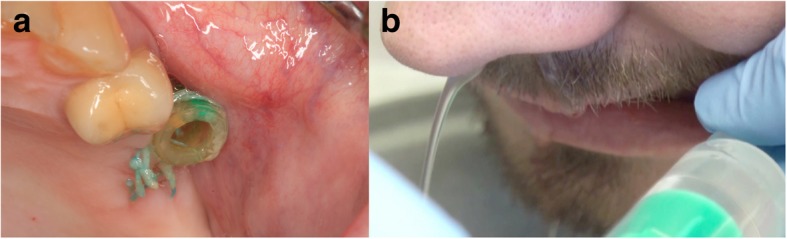
**a** Drainage through the OAC. **b** Irrigation with saline through the OAC

Preoperatively, drainage and irrigation with saline through the OAC of the affected maxillary sinus should be achieved in cases with sinus infection and degenerated mucosa [16]. This procedure should be performed until the lavage fluid is clear and no longer contains inflammatory exudates (Fig. 6a, b). Nasal decongestants shrink the nasal mucosa and keep the antral opening patent for drainage.

Additionally, the use of appropriate antibiotics is necessary prior to surgery.

## Operative procedures

**Fig. 7:**
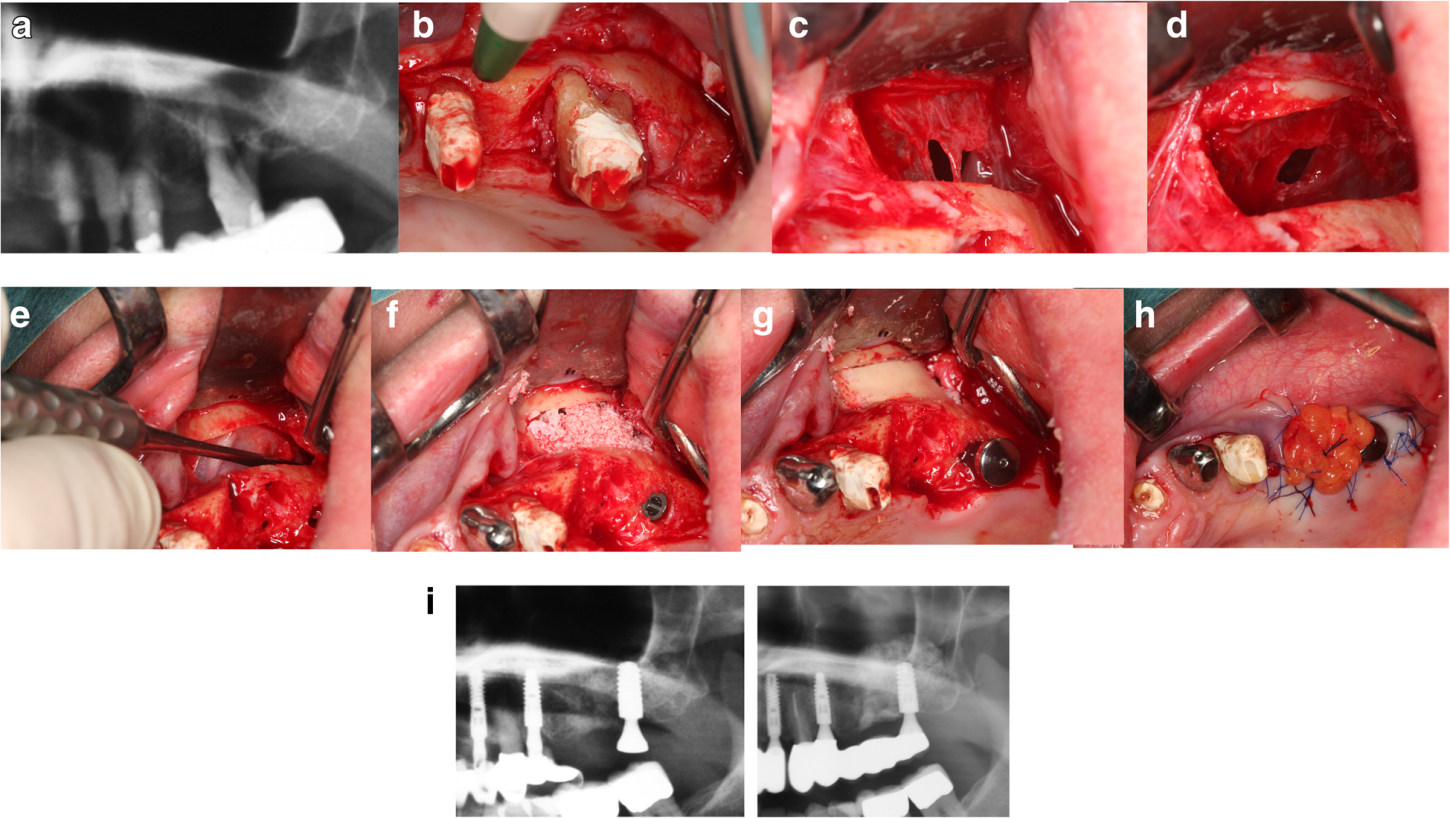
**a** Pre-operative X-ray. **b** Tooth 26. **c** Perforation of the Schneiderian membrane. **d** Perforation after elevating the Schneiderian membrane. **e** Covering the perforation with a collagen membrane and fibrin glue. **f** Augmentation and implant inserted. **g** Repositioning of the buccal bone. **h** Covering of the OAC with the BFP. **i** Post-operative X-ray vs 3 years post

The size of the OAC and opening duration are crucial prognosis factors in treatment. However, primary suturing the gingiva with a figure-of-eight suture closes the communication effectively. When this does not provide adequate closure, a soft tissue closure using a buccal or palatal flap is indicated [22]. It is also possible to close the OAC simultaneous with an immediate implant or to perform an external sinus elevation [23, 24] (Fig. 7 a–i).

Prior to surgical treatment of oroantral communication, a previous diagnostic is achieved to exclude the presence of a foreign body and/or inflammatory changes of the mucous membrane [25]. It is of paramount importance to close the oroantral fistula in a disease-free sinus environment [26].

In case of fully developed fistulae, excision of the fistulous epithelial tract must be achieved, mucosa should be debrided up to the well-perfused tissue, and the infected bony structures should be curetted [27].

**Fig. 8:**
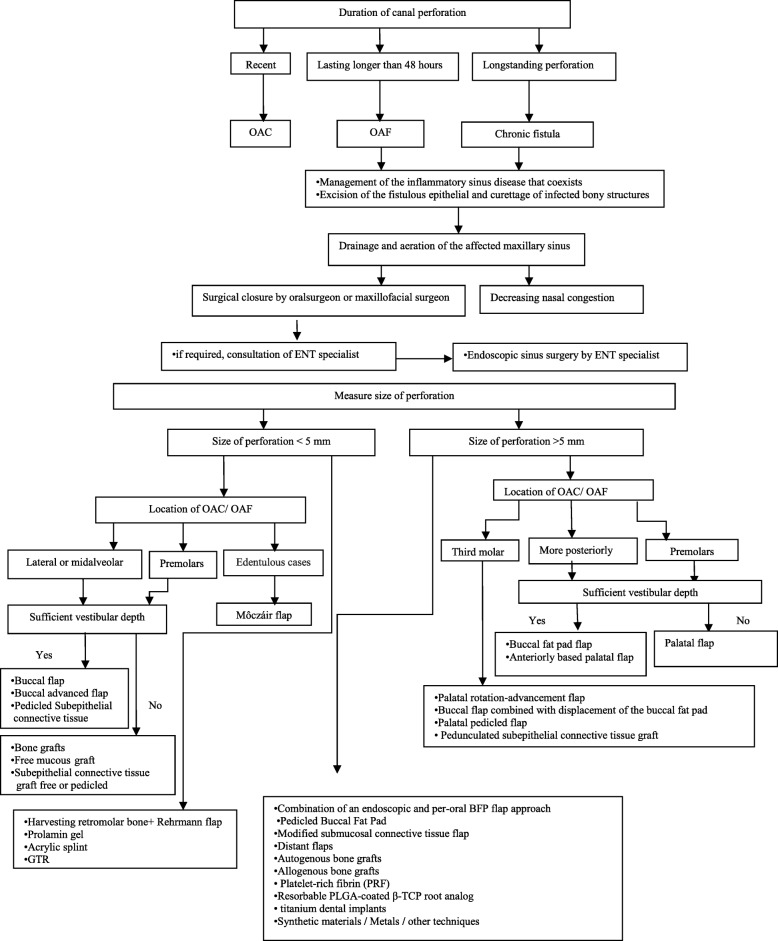
Decision tree for the closure of OAC and OAF including suggested treatment options based upon size, location, and time of diagnosis of OAC and OAF

Many techniques have been described for the closure of oroantral fistula, including local and soft tissue flaps. Other techniques include grafts, allogenous, xenografts, alloplastic materials, and other methods like guided tissue regeneration (GTR) or immediate implantation of a dental implant (Fig. 8) (Table 1).

**Table 1:** Techniques for closure OAC/OAF

Local soft tissue flaps	Buccal flaps	Buccal flap (Rehrmann flap) Môczáir flap Buccal advanced flap Buccal fat pad flap Pedicled buccal fat pad Buccal flap combined with displacement of the buccal fat pad
	Palatal flaps	Palatal flap Palatal rotation-advancement flap Palatal pedicled flap Anteriorly based palatal flap Palatal hinged flap Palatal mucoperiosteal rotation flap Palatal straight advancement flap Palatal pedicled island flap Modified submucosal connective tissue flap Submucosal connective tissue pedicle flap Submucosal island flap Random palatal flap
	Grafts	Free mucous graft Subepithelial connective tissue graft
Autogenous distant flaps		Tongue flap Auricular cartilage Septal cartilage Temporalis muscle flap
Autogenous bone grafts		Intraoral Extraoral
Autogenous fibrin		Platelet-rich fibrin
Allogenous		Fibrin glue Dura
Xenografts		Collagen Gelatin film Bio Gide/Bio Oss
Synthetic materials/metals		Gold Aluminum Tantalum Polymethylmethacrylate Hydroxyapatite Root analogue Titanium dental implant
Other techniques		Tooth transplantation Interseptal alveolotomy Guided tissue regeneration Prolamin gel Splint Biostimulation with laser light

A rational decision-making process has to be followed for the closure of OAC/OAF rather than randomly practicing the available technique. Clinically, the well-perfused flap demonstrates a wider base and is well vascularized. The site of anastomosis should be free of tension and situated over the intact alveolar bone leaving at least 5 mm from the margin of fistula [10].

**Fig. 9:**
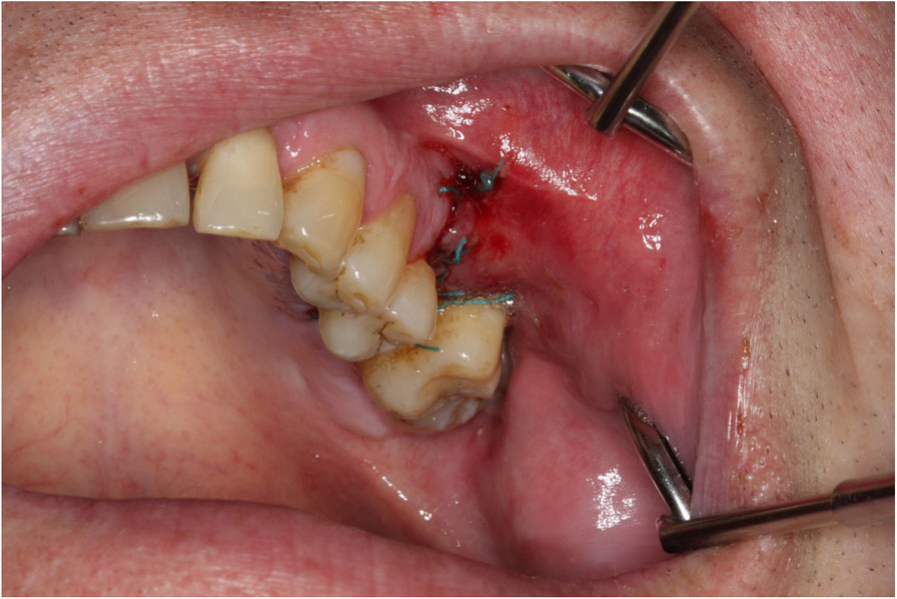
Closure by Rehrmann flap

Local buccal soft tissue flaps are often indicated in the closure of small to moderate size defects [28]. It worth noting that the reduction of buccal vestibular height following the closure by buccal flap (Rehrmann flap) makes it difficult to use prosthesis in the future (Fig. 9). For further implant treatment after performing Rehrmann plasty, it could be necessary to perform an apical reposition flap or an apical reposition flap combined with free gingival graft (FGG) to increase the width of keratinized mucosa. OAF can be closed successfully with the buccal advancement flap in cases where vestibular obliteration will not be a complication [29]. Other options are free gingival grafts (FGG) from the palate or free connective tissue grafts (CTG) in the premolar area or pedicled connective tissue grafts (CTG) in the molar area. These two methods should be preferred in view of later implantation because the depth of the vestibulum remains in the original position. The free mucosal graft is more uncomfortable for the patient due to secondary wound healing.

**Fig. 10:**
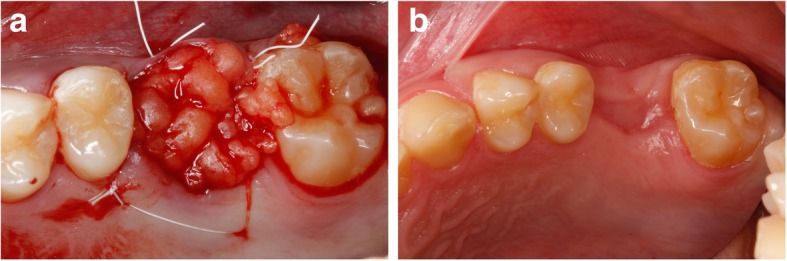
**a** Closure by the buccal fat pad. **b** Healing after 3 months closure by buccal fat pad

Borgonovo et al. proposed the use of the buccal flap for the closure of oroantral fistulae of moderate size, provided that not too posteriorly located; the palatal flap is best used in the case of fistulae located in the premolar teeth area; and the buccal flap combined with displacement of the buccal fat pad (BFP) is appropriate for fistulae located in the third molar area [10]. Ideally, a combination of BFP with buccal advancement flap technique can be used to cover BFP and as additional tissue in cases of deficient BFP for closure (Fig. 10 a, b) [30].

Given the limitations of local flaps option for closure OAF, distant flaps and bone grafts can be used with success in the closure of large defects or in cases where local flaps have failed [28].

Application of alloplastic, biological material, or immediate implantation for the closure of OAC is usually indicated in the closer of OAC with a diameter of 3–4 mm provided that the maxillary sinus is uninfected or no foreign body is within the antrum [31, 23].

**Fig. 11:**
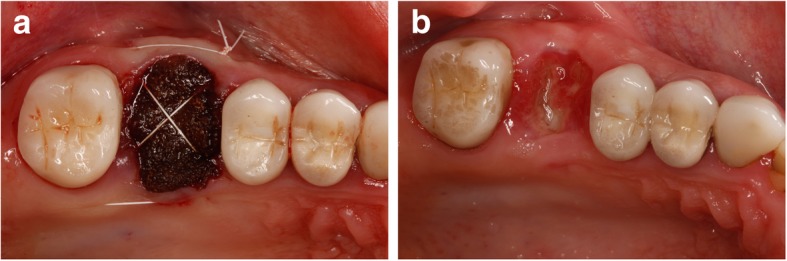
**a** Healing after closure by oxidized cellulose. **b** Healing after 14 days

Among the various synthetic materials, Bio-Oss-Bio-Gide Sandwich technique has yielded excellent results for OAF closure. The technique achieves both bony and soft tissue closure, by contrast with only soft tissue closure obtained by local flaps [30]. Collagen and fibrin materials have received considerable attention for these are biologically competent and easy to use (Fig. 11 a, b) [28].

The failure rate of closure of large oroantral fistulas increases owing to the large defect in the underlying bone that supports the overlying flap [32]. Many techniques are used to reconstruct this bony defect, including metals, autogenous bone grafts, and nonporous hydroxyapatite blocks.

Considering autogenous bone grafts as the technique of choice for closure large OAF, donor site morbidity, anatomic and structural problems, and increased level of bone resorption during healing should be borne in mind [22]. However, bone grafts are recommended for the closure of chronic OAF when soft tissue flap closure fails [33].

It is recommended to use resorbable guided tissue regeneration membrane when endosseous implant with bone graft is considered [34]. More recently, a high-density polytetrafluoroethylene (PTFE) membrane is used to close an OAC. This technique showed a complete closure of the OAF due to the good regeneration of the soft tissues directly over the OAC [35].

Furthermore, single-stage alveolar augmentation with autogenous bone graft and platelet-rich fibrin (PRF) has found its application as a non-invasive contemporary technique for the closure of OAF [25, 36].

Non-surgical closure of OAC with absorbable polyglactin/polydioxanon implant can be applied in higher risk patients with blood disorders [31]. Moreover, an acrylic surgical splint can be used successfully when a surgical intervention is contraindicated because of immunosuppression [1].

## Postoperative procedures

Oral care, a diet of soft foods, analgesics (e.g., non-steroidal anti-inflammatory drugs (NSAIDS)) and nasal decongestants are recommended postoperatively. Further, nose blowing, sneezing with a closed mouth, and vigorous sports should be avoided [12].

## Summary and conclusion

To the authors’ knowledge, decision-making in the closure of OAC and OAF has not been previously reported. With the above mentioned steps, it is possible to close an oroantral communication or fistula by different techniques with particular emphasis on choosing the most relevant technique. A comprehensive clinical and radiographic examination and consideration of the patient history serve to assess the severity of the OAC and the patient’s treatment needs. The criteria of severity of closure of OAF include the size, time of diagnosis of OAF, improper treatment of sinus infection preoperatively, epithelialization of the fistulous tract, and excessive tension on the flap impeding blood supply for healing [37]. Technical criteria of complexity include the location of OAF, quantity and quality of tissue at the site of OAF, size, vestibular depth, and clinical experience. The article first provides a summary of the management considerations and diagnostic modalities for the closure of OAC and OAF and then presents a framework for decision-making in their closure.

## Abbreviations

BFP: Buccal fat pad
CBCT: Cone beam computed tomography
CT: Computed tomography
CTG: Connective tissue grafts
FGG: Free gingival graft
FMG: Free mucosal graft
GTR: Guided tissue regeneration
OAC: Oroantral communication
OAF: Oroantral fistula
PRF: Platelet-rich fibrin
PTFE: Polytetrafluoroethylene
